# Recent morphologic evolution of the German Wadden Sea

**DOI:** 10.1038/s41598-019-45683-1

**Published:** 2019-06-26

**Authors:** Markus Benninghoff, Christian Winter

**Affiliations:** 1 MARUM - Center for Marine Environmental Sciences and Faculty of Geosciences, University of Bremen, Bremen, 28359 Germany; 2 Institute of Geosciences, Christian-Albrechts-Universität zu Kiel, Kiel, 24118 Germany

**Keywords:** Ocean sciences, Geomorphology

## Abstract

The Wadden Sea is a unique and important intertidal coastal zone under the pressure of changing driving forces (i.e. sea level rise, storm surges and increasing tidal range). In this study, we characterize the recent morphologic evolution of the German part of the Wadden Sea for the time period 1998 to 2016 based on a large dataset of available digital elevation models. A sediment budget analysis reveals that the Wadden Sea is accumulating sediment. Changes in the ratio of intertidal to subtidal surface area indicate an extension of the intertidal zone. Most of the intertidal flats accumulate sediments with rates higher than the observed mean sea level rise in the German Bight, while simultaneously the subtidal mean depth increases. For the period of investigation this Wadden Sea steepening is quantified to averaged values of +7.9 mm/yr for the tidal flats and −20.0 mm/yr for the channels.

## Introduction

Situated in the southern North Sea, extending from the Netherlands to Denmark, the intertidal Wadden Sea represents an environment of high importance for local ecology and economy^1^. The German North Sea coastline expands over approximately 300 km, corresponding to about 60% of the total extent of the Wadden Sea. It features a variety of different coastal landscapes (barrier islands, Halligen, sandy beaches, open and back-barrier sand and mud flats, tidal channels, and estuaries), provides habitats for a large range of species^2^, and is driven by diverse morpho- and hydrodynamic conditions^3,4^. The barrier islands and back barrier basins of the Wadden Sea, the open tidal flats and the three major estuaries Ems, Weser, and Elbe are the geomorphologic result of the post-glacial sea level rise in combination with sufficient sediment availability^5,6^. Also, coastal protection measures and land reclamation have changed the coast over the last millennial^7^. The German mainland North Sea coast is almost entirely diked, and locally beach nourishments and river capital and maintenance dredging are undertaken^8,9^ on a regular basis.

The Wadden Sea is a UNESCO World Heritage Site and protected by National Park regulations of three German federal states. At the same time it is of socio-economic importance to the inhabitants and industries of nearby urban areas^7^. As it is a highly dynamic environment at low altitude^10^, exposed to extreme forcings by waves and tides the Wadden Sea system is highly vulnerable to climate change effects like sea level rise and changes in meteorological and hydrodynamic forcing^11^.

**Figure 1:**
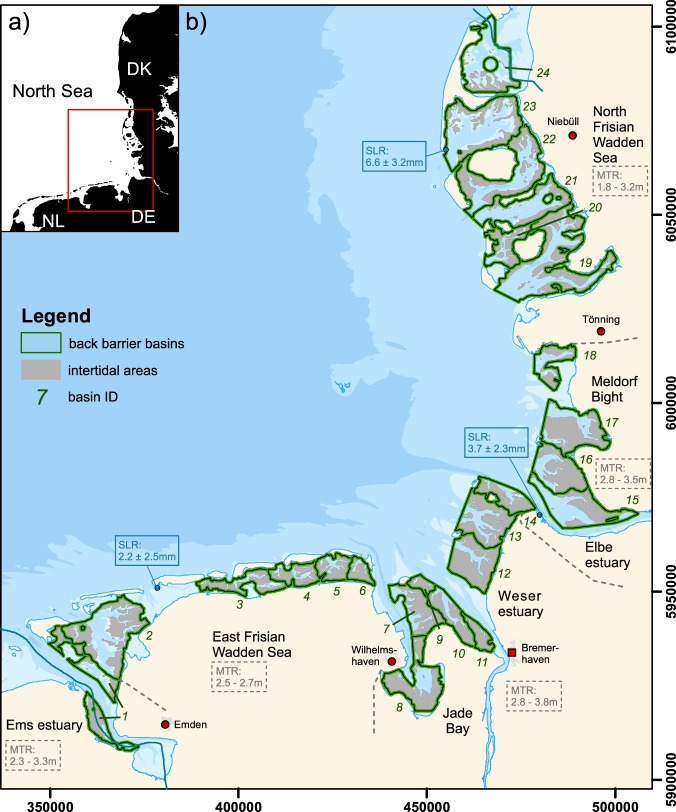
(**a**) Study area German Wadden Sea in the North Sea. (**b**) Map showing the different morphological entities in the German Wadden Sea considered in this study. Green polygons indicate the reference basins, in estuaries, bays and behind back-barrier islands. Grey areas identify intertidal areas, based on an averaged DEM. Relative mean sea level trends (SLR) for the time period 1993–2011 are adopted from Wahl *et al*.^20^ and highlighted at the specific locations. Intervals of mean tidal range (MTR) are determined for the individual regions based on data from the Federal Maritime and Hydrographic Agency (BSH)^53^. The data for the digital elevation model origins from the AufMod project^25^ provided by the BSH. Coastlines^54,55^/city locations^55^ are based on data from the GSHHG data set, and OpenStreetMap. Low water levels (intertidal boundaries) were calculated using the Delft3D suite. The data was processed and the map in this figure was generated using ArcGIS 10.4 and CorelDraw 2017. Projection: WGS 84/UTM zone 32 N.

The German Wadden Sea can be divided into five regions, the outer Ems estuary, the East Frisian Wadden Sea (EFWS), the Jade Bay and outer Weser estuary, the outer Elbe estuary and greater Meldorf Bight area, as well as the North Frisian Wadden Sea (NFWS)(Fig. 1). These regions feature different governing driving forces and geologic-morphological settings. The three major (outer) estuaries exhibit a generally higher tidal range (Fig. 1) and varying riverine freshwater discharge (Ems: 17–360 m³/s (mean: 80 m³/s), Weser: 116–1200 m³/s (mean: 316 m³/s), Elbe: 276–1970 m³/s (mean: 704 m³/s))^12,13^. The region between Weser estuary, Elbe estuary and Meldorf bight features exposed open tidal flats and only smaller, mainly uninhabited islands and banks. The EFWS and NFWS feature barrier islands, tidal channels, and back barrier basins. The intertidal flats mostly consist of fine sand to silt, while the subtidal regions having coarser material^14^.

## Sea level rise and its impact on the Wadden Sea

Tidal flat sedimentology and morphology depend on the Holocene setting and evolution, recent sea-level, wave and tidal forcing, as well as sediment supply^15,16^. Changes in these driving forces lead to the adaptation of tidal flats and their supplying/draining channels. Morphologic response times vary from short term (tidal cycle/storm event), to longer term (months/years)^15,17,18^. In the last decades, an accelerated sea level rise unprecedented for the last 2000 yrs^19^, ranging from 2.2 ± 2.5 to 6.6 ± 3.2 mm/yr, was observed in the German Bight^20^. An analysis of 20 tidal gauges in the German Wadden Sea^21,22^ showed a regional pattern of opposing trends in the development of the tidal range development in the considered time period. The tidal range increased in the Ems and the western part of the EFWS (average: 2.3 mm/yr), as well as slightly in the Outer Weser estuary (average: 0.1 mm/yr), and NFWS (average: 0.1 mm/yr). In contrast to that, the mean tidal range (MTR) decreased in the eastern part of the EFWS and the Jade (average: −1.8 mm/yr), as well as the region between Cuxhaven to Meldorf Bight (average: −2.9 mm/yr). This regional diversity reflects the diverse local morphological and hydrodynamic conditions, and modifications thereof. Corresponding morphologic responses are likely to be expected, as common system understanding assumes continuous development towards new equilibria to given mean sea level and changing tidal properties^11,15,23^.

### Objectives

The accelerated rise of the mean sea level may pose a threat to the stability of the Wadden Sea with its tidal channel-shoal systems^11,24^. It is therefore of significant importance to investigate whether and how the different Wadden Sea elements are keeping up with sea level rise. Although conceptual and numerical models are available, there is a gap of knowledge considering the observed recent morphologic adaption of the German Wadden Sea. In this study, we assess the recent evolution based on a set of annual digital elevation models (DEM) for the period 1998 to 2016 and set the findings into perspective to common schematic models of tidal system morphodynamics.

## Results

Spatial integration of available topographic data^25,26^ from echo sounder and LiDAR measurements allows characterization of the morphologic evolution of many of the German Wadden Sea basins. In the following, analyses describe the evolution between 1998 and 2016, unless stated otherwise. In each basin we differentiate between intertidal (IT) and subtidal (ST) areas (Fig. 1).

### Accretion and erosion rates of individual basins

**Figure 2:**
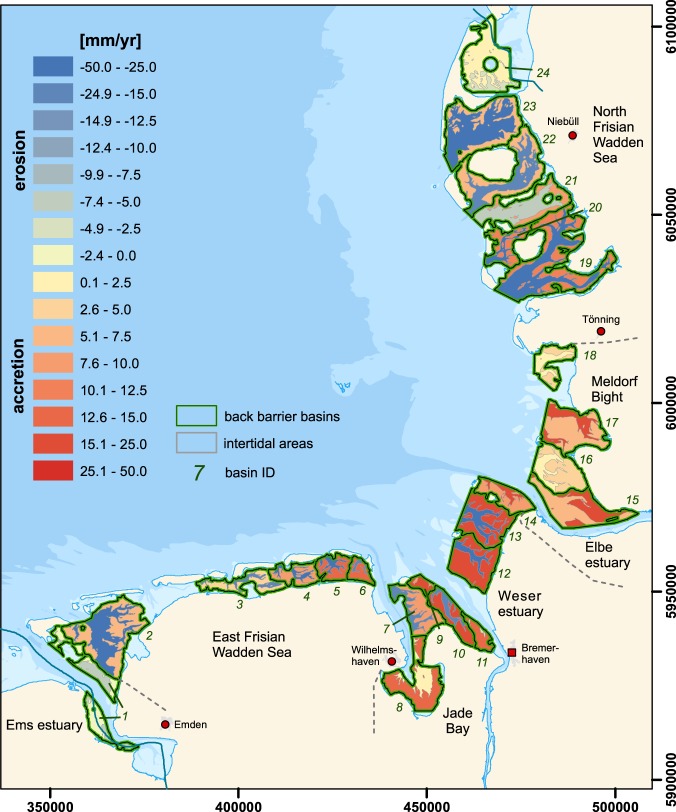
German Wadden Sea basins showing erosion/accretion rates of intertidal and subtidal areas where data was sufficient. The data for the analysis origins from the AufMod project^25^ provided by the Federal Maritime and Hydrographic Agency. Coastlines/city locations are based on data from OpenStreetMap^55^. The data was processed and the map in this figure was generated using ArcGIS 10.4, Matlab 2016b, and CorelDraw 2017. Projection: WGS 84/UTM zone 32 N.

The majority of the different Wadden Sea basins show opposing trends of the subtidal and intertidal evolution: Almost all of the intertidal areas show an accretion of sediments, whereas the majority of subtidal areas deepen over time. The basins of the East Frisian Wadden Sea (ID 2 to 6) show accretion of the intertidal flats in the range of +5 to +14 mm/yr while the mean level of the subtidal areas decreases (−48 to −17mm/yr) (Fig. 2). Five out of seven basins in the Jade-Weser estuary (ID 7 to 13) show a clear trend of subtidal deepening (−37 to −3mm/yr, average: −24mm/yr), whereas two basins, one of which the Jade bay, indicate a decrease in subtidal mean depth (+2 to +8 mm/yr). All basins reveal sedimentation in the intertidal areas (+8 to +22 mm/yr, average: +16 mm/yr).

All basins in the Elbe estuary and Meldorf bight regions (ID 14 to 18) show both accretion in the subtidal and intertidal areas (+2 to +24 mm/yr). The back barrier basins of the North Frisian Wadden Sea (ID 19 to 24) show similar accretion of the intertidal and deepening of the subtidal as the majority of basins in the East Frisian Wadden Sea and Jade-Weser estuary.

### Intertidal areas expand

**Figure 3:**
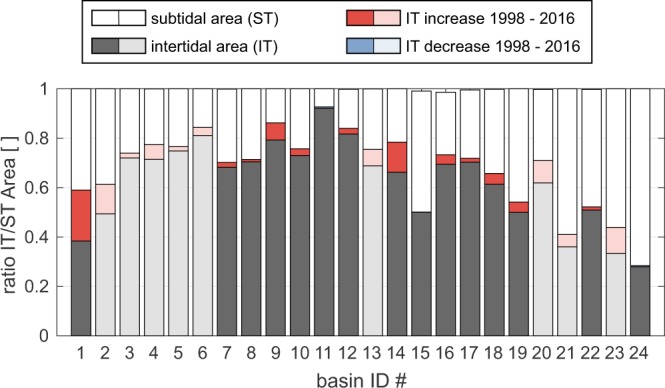
Ratio of intertidal areas and total basin area. Red bars indicate an extension of the intertidal area from the first to the last five years of the investigated period. Blue bars indicate a decrease. The basin IDs correspond to the map in Fig. 2. Lighter colours represent basins with lower data coverage, and potentially higher uncertainty. The data for the analysis origins from the AufMod project^25^ provided by the Federal Maritime and Hydrographic Agency. The data was processed and plotted using ArcGIS 10.4, Matlab R2016b, and CorelDraw 2017.

In the individual basins the fraction of intertidal areas varies from 27 to 92%, the highest values to be found in the East Frisian Wadden Sea and Jade/Weser area (around 70%), and the lowest values in the North Frisian Wadden Sea (below 50%). It is notable that the intertidal area has increased in almost all basins (Fig. 3). Intertidal flats in the West, near the Ems estuary (IDs 1, 2) have extended the most. Here a shift in intertidal to total basin area of more than 18% has occurred, which is due to the degradation of the (former river mouth) Easter Ems^27^. In contrast to the Ems estuary, the intertidal area in the Weser estuary (ID 7 to 13) has only changed by −1 to +7%, whereas the intertidal areas next to the Elbe River (ID 14 to 16) show an increase between 0 to +12%. The proportional gain of the remaining basins can be considered almost uniform around +5%. For all analysed basins that results in an increase of 57% of the total area in the first four years of the investigated period (1998–2002) to 62% in the last four years (2012–2016) with the according reduction of the subtidal area.

### Sediment budgets of individual basins

**Figure 4:**
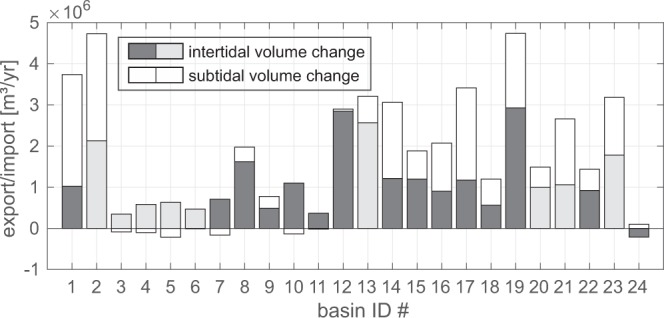
Import and export trends per basin divided into intertidal and subtidal. Intertidal import/export is defined as the rate of change of sediment volume above MLW. Subtidal import/export as the change of (negative) water volume below MLW. Lighter colours indicate basins with a lower data coverage, and potentially higher uncertainty. The data for the analysis origins from the AufMod project^25^ provided by the Federal Maritime and Hydrographic Agency. The data was processed and plotted using ArcGIS 10.4, Matlab R2016b, and CorelDraw 2017.

The increase of the horizontal extent and vertical growth of the intertidal implies a gain of sediment in the intertidal areas. Here, we define the intertidal sediment volume (ISV) as the volume of sediment in between mean high water level (MHW) and mean low water level (MLW), and the subtidal channel volume as the water body below MLW. The total gains and losses broken down to the single basins suggest potential exchange of sediment between the subtidal and the intertidal regions, or elsewhere (Fig. 4). The overall gain is generally higher near the Ems estuary (ID 1, 2), as well as north of the Weser estuary to lower North Frisian Islands (ID 12–19). The remaining North Frisian Wadden Sea (ID 20–24), the East Frisian Wadden Sea and large areas of the Jade/Weser estuary show lower values.

Five basins in the EFWS (ID 3–5), and the Jade/Weser estuary (ID 7, 10) show a loss of sediment in the subtidal and thus may hint at a potential redistribution of sediment to the intertidal. However, the intertidal gains exceed the losses from the subtidal areas. Only one basin (ID 24) exhibits intertidal sediment loss. As 23 out of 24 basins show a positive overall budget, an overall import of sediment is implied.

It should be noted that for some basins the indicated subtidal import of sediment seems to be in conflict with the overall deepening trends observed in the map (Fig. 2). However, the import of sediment to the subtidal areas is also related to the extension of the intertidal areas (Fig. 3) and is indicative for an overall steepening of the channel shoal system.

### Overall sediment budget

Analyses indicate a total net sediment turnover of 597 Mm³ for the considered 24 basins (Table 1). The intertidal areas gained 352 Mm³ (+20.4%). The total subtidal channel volume has reduced by 245 Mm³ (5.6%), indicating that intertidal gain cannot be compensated by subtidal deepening. Furthermore, the decrease in subtidal channel volume, paired with the increase in mean depth (Fig. 1), implies that the system tends towards narrower and deeper channels.

**Table 1:** Import of sediment, derived on the basis of changes in Intertidal Sediment Volume (ISV) and (negative) Subtidal Channel Volume (SCV) for the five regions in the German Bight.

	avg. Volume 1998–2002 [10^6^ m^3^]		avg. Volume 2012–2016 [10^6^ m^3^]		Sediment Import/Export [10^6^ m^3^]		Volume change [%]	
	ISV	*SCV*	ISV	*SCV*	IT	*ST*	ISV	*SCV*
Ems estuary	32.8	−*146.6*	48.4	−*104.1*	+17.8	+*42.5*	+58.0%	−*29.0%*
East Frisian Wadden Sea*	197.2	−*443.4*	253.9	−*414.3*	+56.7	+*29.0*	+28.8%	−*6.6%*
Jade/Weser estuary	606.5	−*428.4*	727.7	−*419.4*	+121.2	+*9.0*	+20.0%	−*2.1%*
Elbe estuary/Meldorf Bight	513.2	−*679.2*	582.2	−*597.4*	+68.3	+*81.8*	+13.3%	−*12.0%*
North Frisian Wadden Sea	371.5	−*2705.7*	466.9	−*2618.8*	+95.5	+*86.9*	+25.7%	−*3.2%*
***total***	***1724.6***	**−** ***4394.7***	***2076.1***	**−** ***4149.3***	**+351.5**	**+** ***245.4***	**+20.4%**	**−** ***5.6%***

Volumes were averaged for the first and last 5 DEM. Indicated (*) are regions with a lower coverage, and potentially higher uncertainty. The analysed data was provided by the Federal Maritime and Hydrographic Agency^25^. The data was processed and plotted using ArcGIS 10.4 and Matlab R2016b.

## Discussion

The recent decadal morphologic evolution of the German Wadden Sea has been shown based on a compilation of all digitally available bathymetric datasets^25^. A linear trend analysis and volumetric comparisons of intertidal to subtidal areas in 24 basins revealed how the Wadden Sea basins develop towards depositional intertidal and erosional subtidal trends. Generally, tidal flat accretion is assumed to be driven by the observed increase in mean sea level (MSL), which induces feedback mechanisms in the Wadden Sea tidal channel/flat hydro- and morphodynamics^15^: An increase in the accommodation space over tidal flats leads to an increase in time of tidal inundation, favouring the settling of fine suspended sediment. Furthermore, the increase in accommodation space and tidal prism leads to an amplification of tide-induced currents in the tidal channels, thereby enhancing transport capacity. In the case of a more pronounced flood dominance this results in sediment export to the intertidal areas and with it an increase in height (and expansion) of the flats until a new dynamic equilibrium is established, constituting a negative feedback loop^15,28,29^.

In line with these concepts, we see accretion of the intertidal areas in a majority of basins (range: −4 to 22 mm/yr, detailed breakdown available in supplementary Tab. A-2), but with rates higher than the observed mean sea level rise^20^. A majority of basins also feature erosion in the subtidal regions, thus confirming a conceptual model outlined by Hofstede^28^. The observed extension of intertidal flats was conceptualized and explained by Dieckmann^30^ and Friedrichs^15^ to be due to an increase in mean tidal range combined with sediment abundance^31^. The strong shift in subtidal to intertidal area ratio in Ems region basins (1 and 2) has been described by Schubert^27^ as a silting of the former river mouth (Easter Ems channel).

The intertidal areas accrete with rates ranging from 4 to 22 mm/yr. These rates exceed the observed recent mean sea level rise of 2.2 to 6.6 mm/yr for the German Bight^20^. This is in line with De Vet *et al*.^32^ who conducted research on the recent morphologic evolution of tidal flats in the Eastern and Western Scheldt estuary, and found accretion rates of similar order for one of the estuaries (approx. 13 mm/yr). Also Elias *et al*.^1^ investigated the long term sediment transport volumes in the Dutch Wadden Sea for individual basins, which showed an accumulation of sediment, in a similar order of magnitude.

Obviously the rise in mean sea level cannot be assumed as the only forcing factor, rather a non-linear morphologic response to combined mean sea level, tides and wave forcing must be assumed. Higher tidal ranges are likely to induce a steepening of the slope^15^, as it was observed in our study area. In that line, changes in tidal constituents have been reported, with amplitudes showing a cyclic/decadal behavior^33^. The observed M2, S2 and N2 amplitude in Cuxhaven increased from the late 1990s until the end of the investigation period in 2008^33^. An analysis of 20 tidal gauges in the German Bight confirmed this trend, but showed a decrease of tidal amplitudes (M2, S2) after 2009^21,22^. Despite the clear trend in amplitudes, yearly averaged (spring) mean high water levels did not show a significant positive linear trend^21,22^ (see supplementary Figs A-1, A-2 and Tab. A-1). The connection between tidal properties and morphologic evolution needs further investigation for the German Bight.

Furthermore, a decline in storm index was reported for the North Sea, from 1998 to 2007 (end of investigation period)^34,35^. Low wave energy would constitute an explanation for the observed intertidal sediment accretion, due to limited wave induced erosion^15^. However, an analysis of satellite-derived significant wave heights^36^ for the period from 1998 to 2016 did not confirm such negative trend, but rather a slight increase in winter mean wave heights. The exact mechanisms explaining the observed changes of the German Wadden Sea thus remain to be explained.

### Sediment import sufficient

The capability of tidal flats to rise with the sea level depends on the availability of sediment^37^. In addition, the extension of intertidal areas also requires a supplementary amount of sediment. The observed deposition on, and extension of, tidal flats indicate that for the observation period enough sediment was available, and that the basins were capable to receive a sufficient amount. In total it calculates to a sediment volume of 597 Mm³, which must have been imported into the 24 considered basins in the considered time period. This is about twice the amount of what would scale linearly relative to the sea level. The sediment import to the intertidal areas is higher than the export from subtidal regions. Even though subtidal channels import sediment, imposing a shrinking of the channel volume, the mean subtidal depth of a majority of basins increases, which indicates that channels are steepening.

Sources and pathways of sediments cannot be derived from the available data and thus the origin of sediments remains unclear. Previous studies based on numerical model simulations of the Dutch Wadden Sea^23^ assume that the ebb-tidal delta volume is providing sediment for back barrier growth. Others showed that an expansion of ebb-tidal deltas is also possible with sea level rise^38^. Long-term observations of the Dutch Wadden Sea have revealed a decrease in ebb-tidal delta volume of around 400 Mm³ over the course of 70 years^1^. In our study we show that the EFWS and NFWS back-barrier regions alone demand around 270 Mm³ in a time span of 18 years, with the back-barrier regions being roughly the same size. This constitutes a factor 3 in terms of yearly sediment volume import. Unfortunately data availability does not allow for a budgeting of the foreshore and the highly dynamic delta regions in the German Wadden Sea, but it appears unlikely that the large volume of sediment imported to the back barrier regions originate from the ebb-tidal deltas alone. To elaborate on these correlations and assumptions, further studies on the hydrodynamics and sediment pathways^39^ are required.

Although data on the sediment distribution of the German Wadden Sea is available^14^, the data does neither allow for an assessment of changes in the grain size distribution over time^40^, nor is information available that describe the characteristics of sediment that was imported to the single basins in the past two decades. However, this information is crucial, as sediment availability and characteristics change the morphologic response and response times^15,41–43^.

### Future development

We observed a steepening in the majority of basins, composed of intertidal flat accretion and simultaneous expansion on one side, as well as subtidal channel deepening on the other side. Both developments influence the propagation of the tidal wave^44,45^, tidal properties, and future extreme water levels. In return, these will affect the sediment transport in the Wadden Sea and the individual basins^11,15^.

We point out that the aforementioned process of intertidal/subtidal steepening cannot be assumed to have a linear trend. The accretion of sediment depends on the accommodation space, which decreases with sediment deposition being higher than the rates of sea level rise^11,37^. The reduced accommodation space and time of tidal inundation will oppose sediment deposition on the flats. Nevertheless, the reduction in subtidal channel volume (through intertidal area expansion) can lead to an increase in bedload transport, as tide-induced currents increase. Here, the direction of bedload transport depends on a possible flood or ebb dominance. The lack of data on the hydrodynamics of the individual tidal basins prohibits further statements.

The time span considered in this study is 18 years and does not allow long-term hindcasts, or even prediction of future morphologic evolution, due to the diversity and complexity of the Wadden Sea system. We do not address other forcings or cyclicity eventually affecting sediment deposition or erosion e.g. river runoff, storminess^35^, 18.6 yr lunar-nodal cycle^46^. Fluctuation in sediment accretion over a long term^32^, as well as seasonal tidal flat accretion^47^ have been reported before.

Previous studies in the region have shown the extension and rise of intertidal flats by means of hypsometric analysis^48^, describing differences in the relation between wetted surface area over depth, pointing out that especially the higher-up flats are expanding in size. Pye and Blott^31^ predicted a reduction of intertidal areas, which is due to a landward restrictions resulting from coastal protection measures, eventually leading to a possible drowning with a rising sea level and sediment scarcity.

We acknowledge that this study does not focus on the recent anthropogenic influences in the German Bight. Direct and indirect effects of maintenance dredging, river deepening and dike construction on the evolution of the Wadden Sea are not considered. The results of this study may be affected by data scarcity and uncertainty, an overview of which is explained in the methods section and the supplementary information. Regardless, it is the first approach of a quantification of recent Wadden Sea development based on all available bathymetric data of the area.

## Methods

The main data source for this study is a set of publicly available digital elevation models (DEM), available in an annual resolution for the period of 1998 to 2016. The data covers the German Bight in a 50 × 50 m grid^25^. To derive a consistent dataset on a year-to-year basis, data from multiple measuring campaigns were merged and interpolated by spatio-temporal interpolation. The compilation includes data from shipborne echo sounder, LiDAR and profile measurement surveys, with a highly varying density of measuring points. Given the case that at some locations no annual datasets were available for the respective year, the nearest data points (in space and time) were used for interpolation (method explained in detail by Milbradt *et al*.^26^). The spatio-temporal difference between measurements varies: regions of economic importance (shipping channels) are surveyed more frequently than less accessible areas (tidal flats). The uncertainty of the measurements used to create the individual DEMs is not exactly assessed, but estimated to be 20 cm on average^25^ for the Wadden Sea basins and assumed to be higher in the foreshore areas^49,50^. The dataset underlies restrictions and uncertainties as it is combined of multiple (about 20.000) measuring campaigns. The relatively coarse resolution of 50 × 50 m goes along with a smoothing of morphological features such as small tidal creeks in tidal flats through averaging. The discretization of the morphology may invoke uncertainties. However, the large number of grid cells, as well as the large area considered, are reducing these uncertainties subsequently.

For the identification of tidal flats and channels, a watershed/basin analysis was performed in ArcGIS based on an average DEM, which included all annual datasets for the time period. Smaller basins and artefacts resulting from the application of the method were combined to larger entities. Barrier Islands and topographic features were used as a seaward boundary, where applicable. An inward boundary 200 m from artificial structures, such as dikes, groynes, cables, pipelines, navigational channels, as well as dredge/dumping sites was set. The watershed method is usually applied on terrestrial DEMs with unidirectional water flow. The resulting watersheds were created semi-automatically, and the hydrodynamic watersheds may be at a different location. Watersheds cannot be considered static over the time interval^51^. Still, as the analysis showed, neighbouring basins have a similar behaviour in terms of accretion and erosion patterns and scales, hence not affecting the general statements of this study.

In order to define MLW and MHW levels for the entire German Wadden Sea a hydrodynamic numerical model was applied^40,52^. The model was run for the first half of the year 2012. The resulting MLW values for subtidal regions were inter- and extrapolated for each (nearby) basin, and combined with the 10 year average MLW values from available gauges in the German Bight^53^.

Averaged MLW of each basin were applied on the annual spatio-temporally interpolated DEMs to calculate the intertidal area, intertidal sediment volume, subtidal area and subtidal channel volume of the first and last 5 years (1998–2002 and 2012–2016). When possible, only DEMs with an annual surveyed-to-total basin coverage of more than 33% were used, otherwise we stated that calculations were performed with a lower coverage (indicated by brighter colours in Figs 3, 4). We argue that a minimum coverage of 33% provides a sufficient amount of accuracy, without being too uneconomic.

For better comparison, volumes were calculated based on a fixed MLW level. If we consider a rise in MLW over the 18-year period, the volumes and rates will be lower, as by definition the sediment volume will decrease linearly with time. Still, the rise in MLW is smaller than SLR, and thereby does not affect the general statement of this study.

Besides volumes and surface areas, the mean intertidal height and mean subtidal depth were calculated for each year. A linear regression analysis was applied to the time series of volumes and average heights to calculate export/import rates (Fig. 3), as well as accretion/erosion rates (Fig. 2). To weaken the impact of the spatio-temporal interpolation on calculated trends, different weights were used for the individual values. We used a multiplier, based on the coverage (rounded up first decimal) times a factor 10, to increase the number of points in time. That means that, for the calculation of trends, an annual dataset with >90% coverage weighs twice as much as a dataset surveyed only 41 to 50%, and ten times as much as a dataset, which is almost entirely interpolated (<10%).

The short time series and data scarcity does not justify fitting of a more complex than linear model. We must assume that tidal flat accretion/erosion and subtidal deepening/shallowing is a continuous and presumably linear process. Non-linear evolution, such as the migration of small gullies and creeks, or atmospheric/astronomical cyclicity will affect and weaken the given trends. Although difficult to assess, we assume the results have a higher reliability than simple DEMs of Difference, as outliers and seasonal effects are diminished. We used 20 cm as the uncertainty of each annual dataset and displayed the error bars in the individual plots (see supplementary Tab. A-2). It is obvious that the year-to-year variance is lower than the given range. Hence, we argue that the uncertainty of the individual DEMs is lower, and further reduced by using the linear trends.

Despite the data seems plausible and reveals similarities to Dutch investigations, the East Frisian and the North Frisian Wadden Sea lack a significant amount of data. Areas with less than three annual datasets were not taken into account. The coverage per basin and year is given in the supplementary information. The absolute uncertainty of the data induced by the measurements, positioning and spatio-temporal interpolation on the final subtidal accretion rates cannot be assessed, thus this study must be read as a best guess indicative analysis with a plausible result.

## Supplementary information

Supplementary Data to Recent morphologic evolution of the German Wadden Sea

## Data Availability

Relevant data will be made available on PANGAEA.
