# Supplementary material for: Recent morphologic evolution of the German Wadden Sea
Source: Sci Rep. 2019 Jun 26;9:9293. doi: 10.1038/s41598-019-45683-1 (PMC6594939; doi:10.1038/s41598-019-45683-1)
Supplement: Supplementary file 1 — Supplementary Data to Recent morphologic evolution of the German Wadden Sea [file 41598_2019_45683_MOESM1_ESM.pdf]

Supplementary Data to Recent morphologic evolution of the German Wadden Sea

Authors: Markus Benninghoff*[1] and Christian Winter[2]

1University of Bremen, MARUM – Center for Marine Environmental Sciences, Bremen, 28359, Germany
2Institute of Geosciences, Christian-Albrechts-Universität zu Kiel, Kiel, 24118, Germany
*mbenninghoff@marum.de

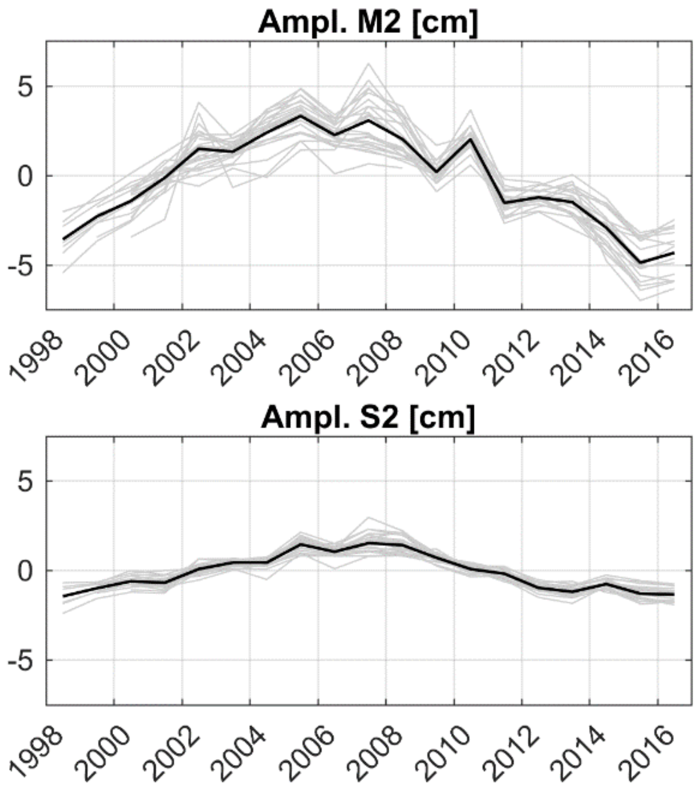

Figure A-1. Yearly average variance of the M2 and S2 amplitude for the considered time period. Amplitudes are calculated for the full year. The analysis is based on waterlevels (temporal resolution: 1 min) provided by the BAFG[25] and the Portal Nordseeküste[26].The data was processed and plotted using Matlab R2016b.

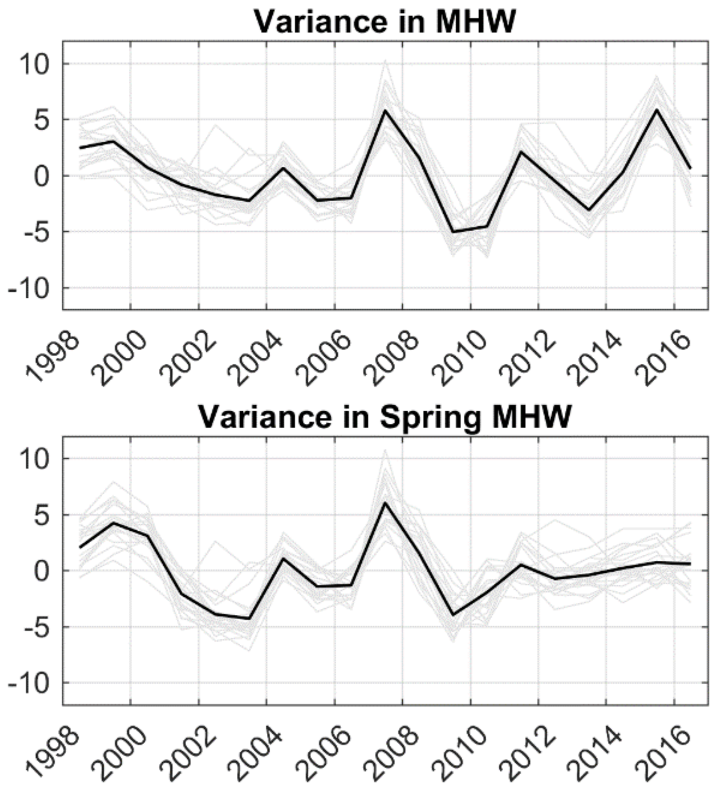

Figure A-2. Changes in MHW and MHW (spring tide only) averaged for 20 tidal gauges in the German Bight. The analysis is based on MHW values provided by the BAFG[25] and the Portal Nordseeküste[26]. The data was processed and plotted using Matlab R2016b.

Table A-1 Average trends of MHW [A] and spring MHW [SP]. Given is also the standard deviation, the min/max, as well as the number of gauges considered in this analysis. The trends are based on an analysis of MHW values provided by the BAFG[25] and the Portal Nordseeküste[26].

		EMS		EFWS		JWE		EMB		NFWS	
full/spring	[F]/[SP]	F	SP	F	SP	F	SP	F	SP	F	SP
avg. inc.	[mm/yr]	0.1	-1.2	1.4	0.4	0.8	0.0	-0.3	-1.3	0.6	0.0
std.dev.	[mm/yr]	0.5	0.5	2.5	2.4	1.4	1.5	1.4	1.2	1.2	1.4
min	[mm/yr]	-0.7	-1.8	-0.3	-1.4	-1.3	-1.9	-1.0	-2.4	-1.2	-1.9
max	[mm/yr]	0.5	-0.9	3.2	2.1	2.3	1.9	1.8	0.4	2.0	1.8
count		3		2		5		4		6	

Table A-2. Changes in intertidal (subtidal) sediment (channel) volume, mean height (depth), and area for each basin/region. The greyscale of the dots and error bars indicate the coverage (and thereby the uncertainty) of the data. Red lines indicate linear fit to the data based on the weights/coverage, rates are given in the individual figures. The data for the analysis origins from the AufMod project[15] provided by the Federal Maritime and Hydrographic Agency. The data was processed and plotted, using ArcGIS 10.4, Matlab R2016b, and CorelDraw 2017.

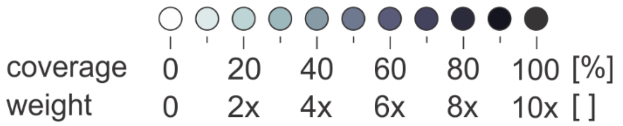

ID		Intertidal Sediment Volume	Intertidal Mean Height	Intertidal Area (neg. ST Area)	Subtidal Channel Volume (*-1)	Subtidal Mean Depth
1	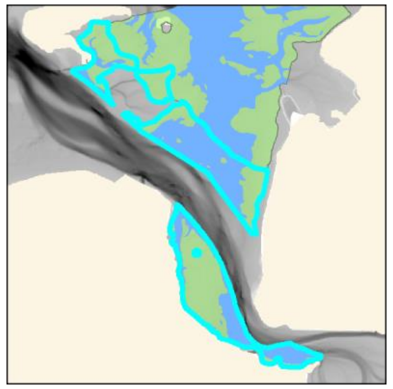	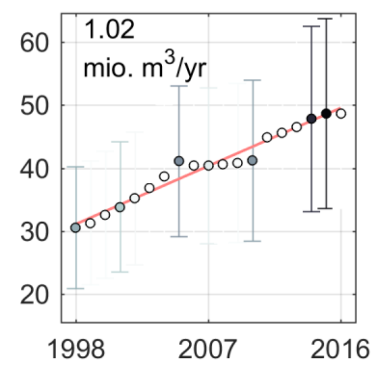	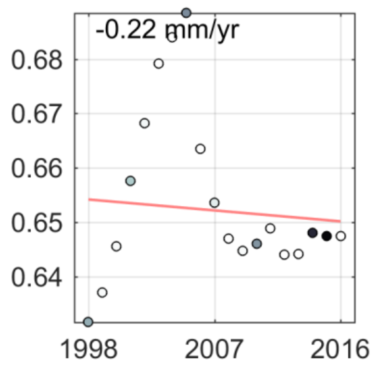	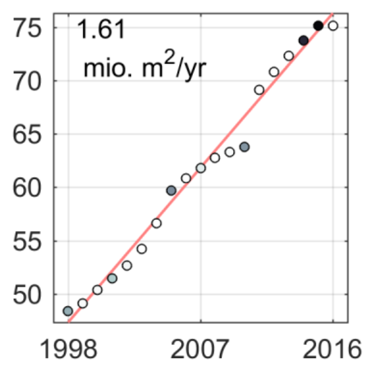	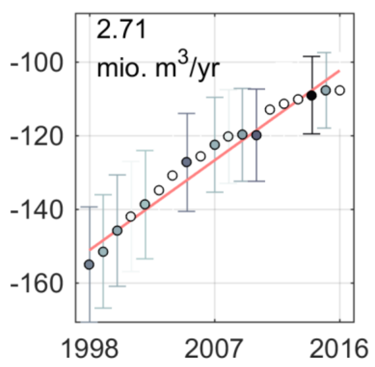	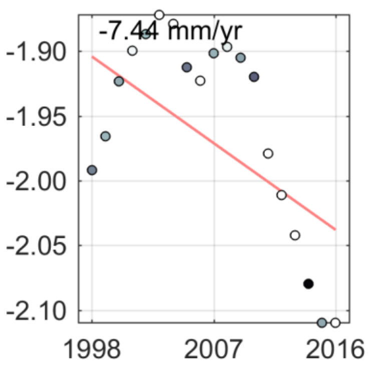
2	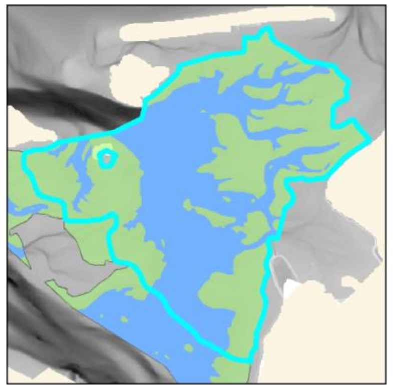	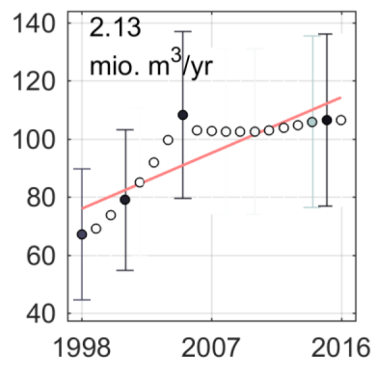	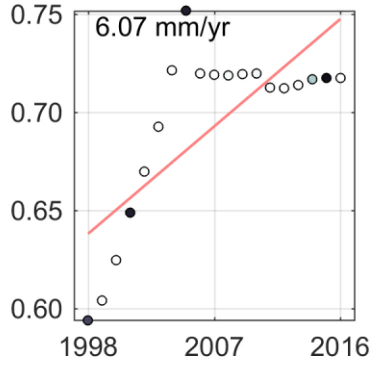	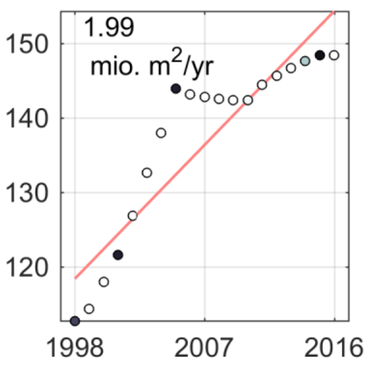	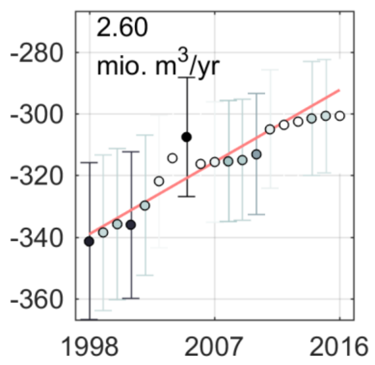	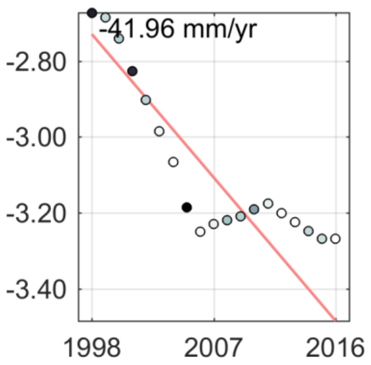
3	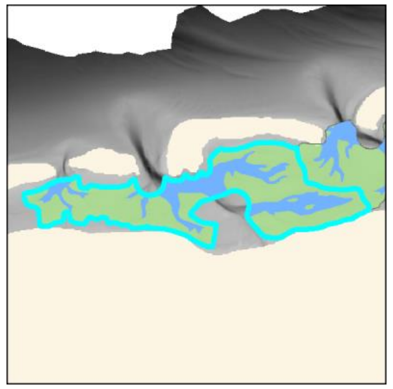	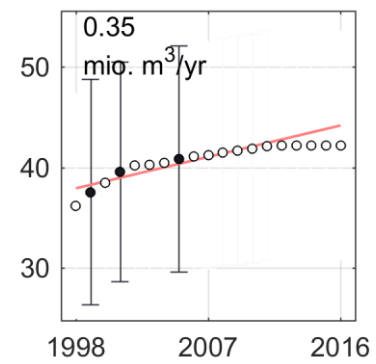	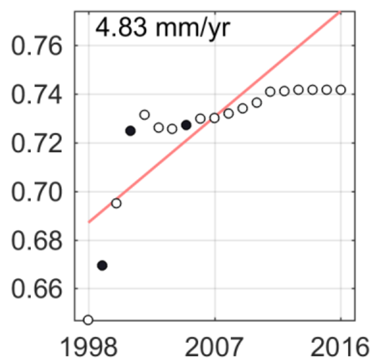	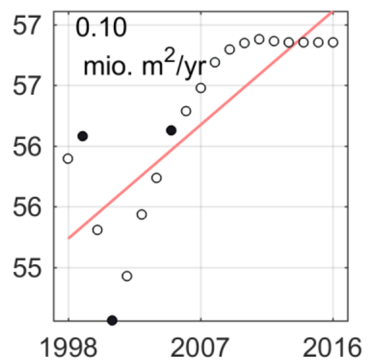	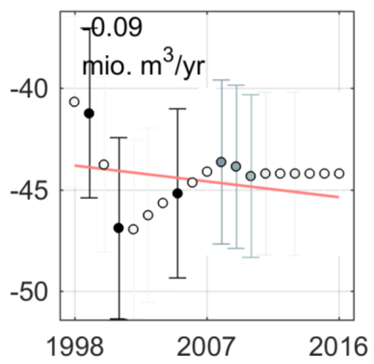	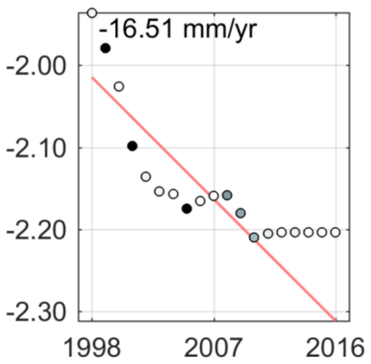
4	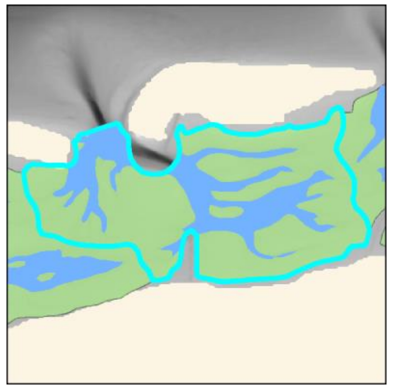	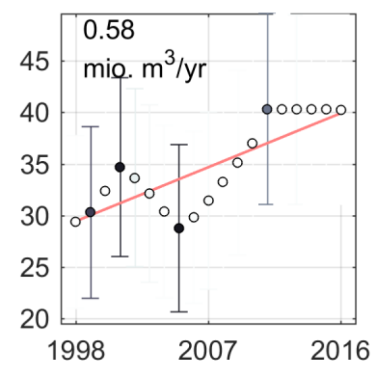	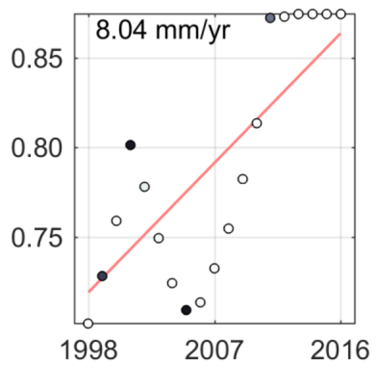	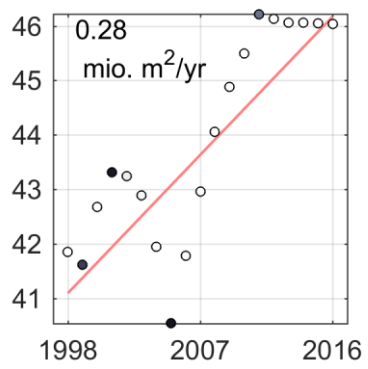	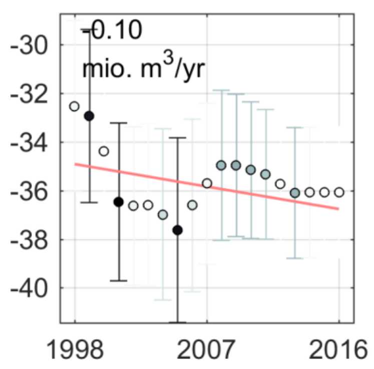	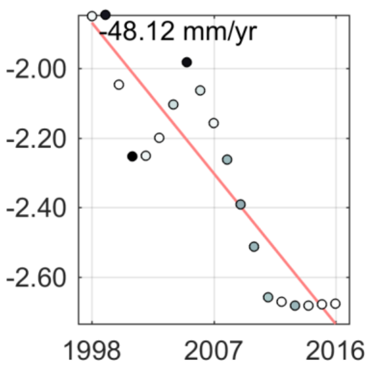

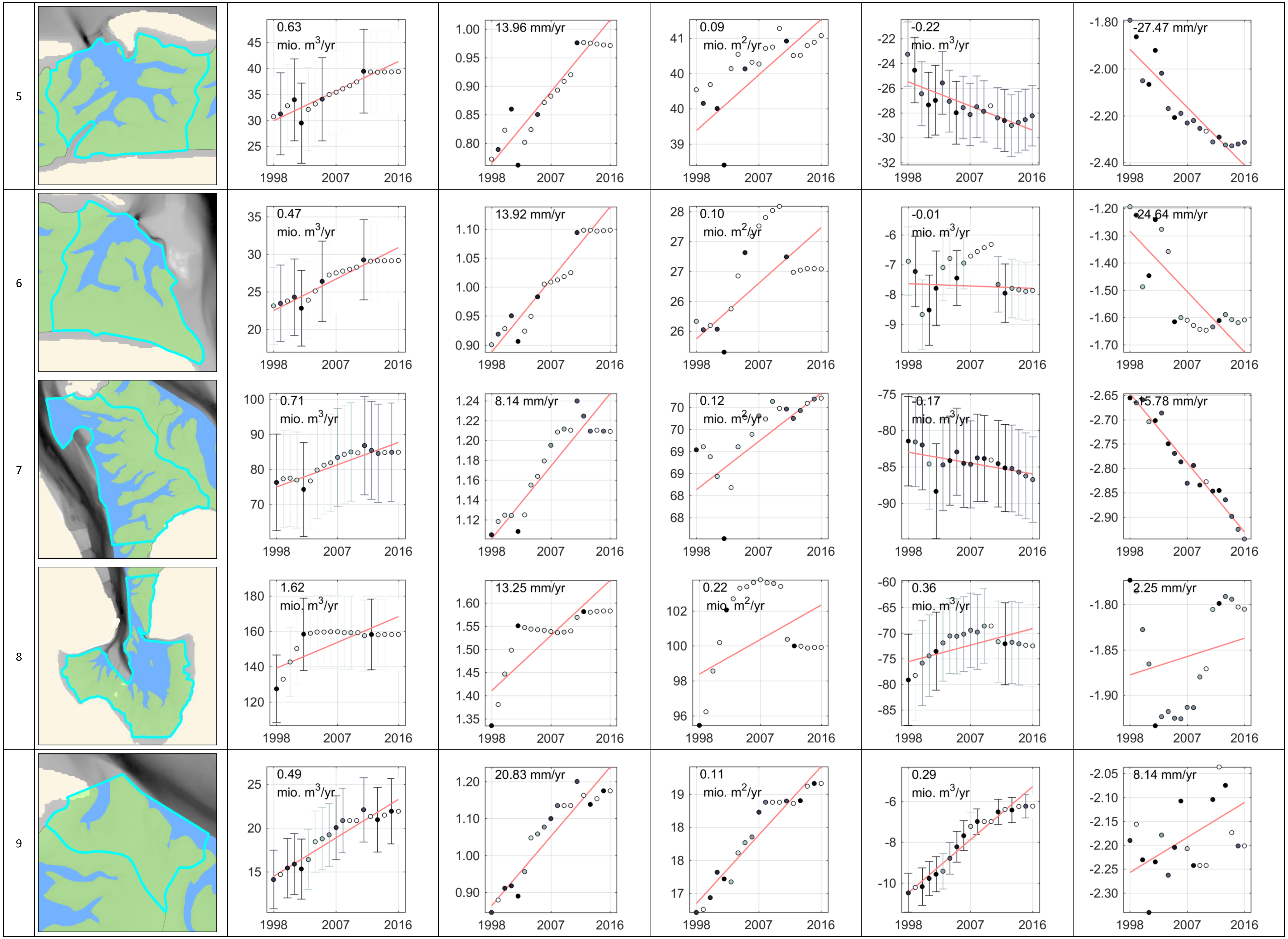

5
0.63 mio. m³/yr
13.96 mm/yr
0.09 mio. m²/yr
-0.22 mio. m³/yr
-27.47 mm/yr
6
0.47 mio. m³/yr
13.92 mm/yr
0.10 mio. m²/yr
-0.01 mio. m³/yr
-24.64 mm/yr
7
0.71 mio. m³/yr
8.14 mm/yr
0.12 mio. m²/yr
-0.17 mio. m³/yr
-15.78 mm/yr
8
1.62 mio. m³/yr
13.25 mm/yr
0.22 mio. m²/yr
0.36 mio. m³/yr
2.25 mm/yr
9
0.49 mio. m³/yr
20.83 mm/yr
0.11 mio. m²/yr
0.29 mio. m³/yr
8.14 mm/yr
1998
2007
2016

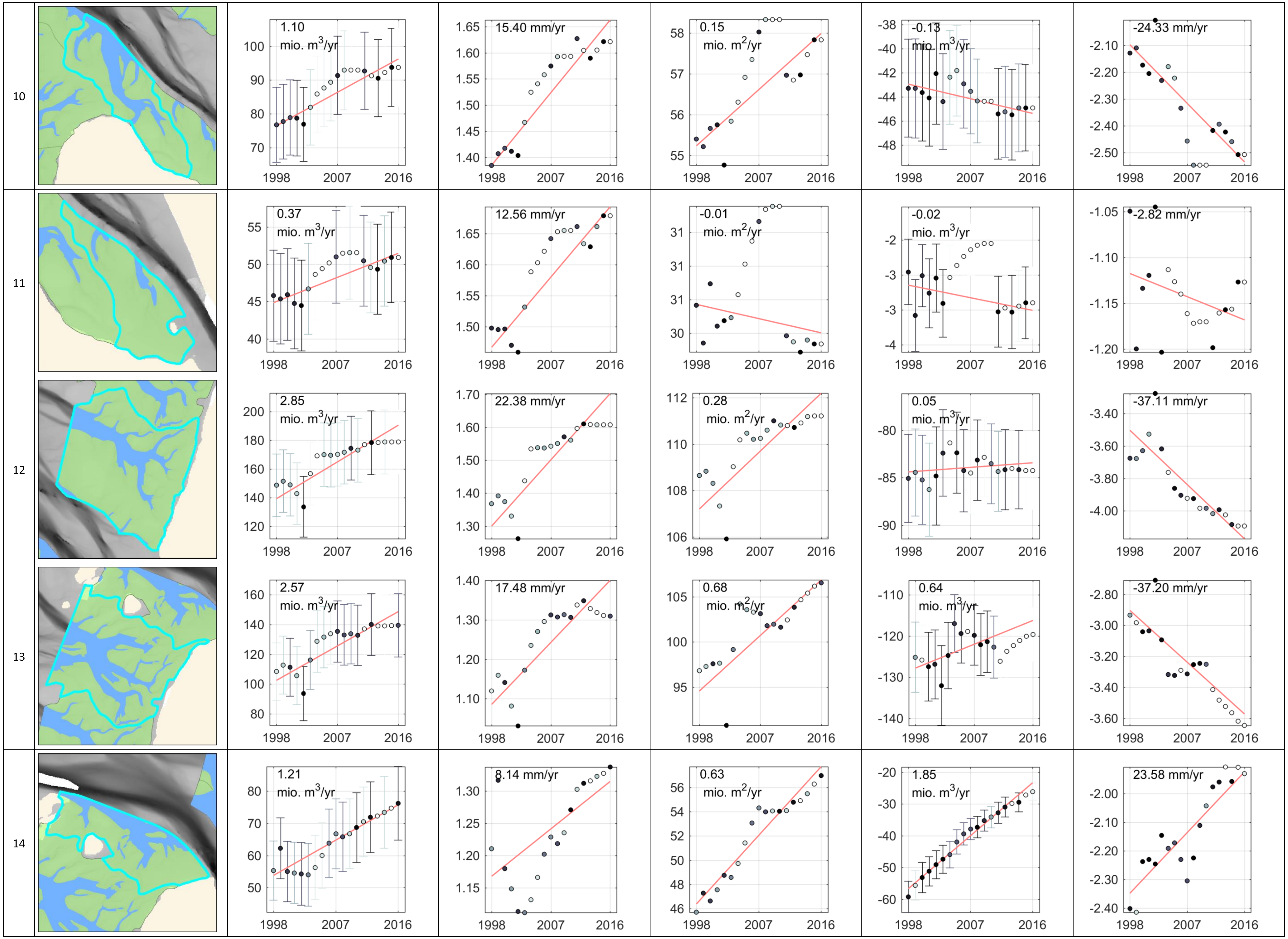
10
1.10 mio. m³/yr
15.40 mm/yr
0.15 mio. m²/yr
-0.13 mio. m³/yr
-24.33 mm/yr
11
0.37 mio. m³/yr
12.56 mm/yr
-0.01 mio. m²/yr
-0.02 mio. m³/yr
-2.82 mm/yr
12
2.85 mio. m³/yr
22.38 mm/yr
0.28 mio. m²/yr
0.05 mio. m³/yr
-37.11 mm/yr
13
2.57 mio. m³/yr
17.48 mm/yr
0.68 mio. m²/yr
0.64 mio. m³/yr
-37.20 mm/yr
14
1.21 mio. m³/yr
8.14 mm/yr
0.63 mio. m²/yr
1.85 mio. m³/yr
23.58 mm/yr
1998
2007
2016

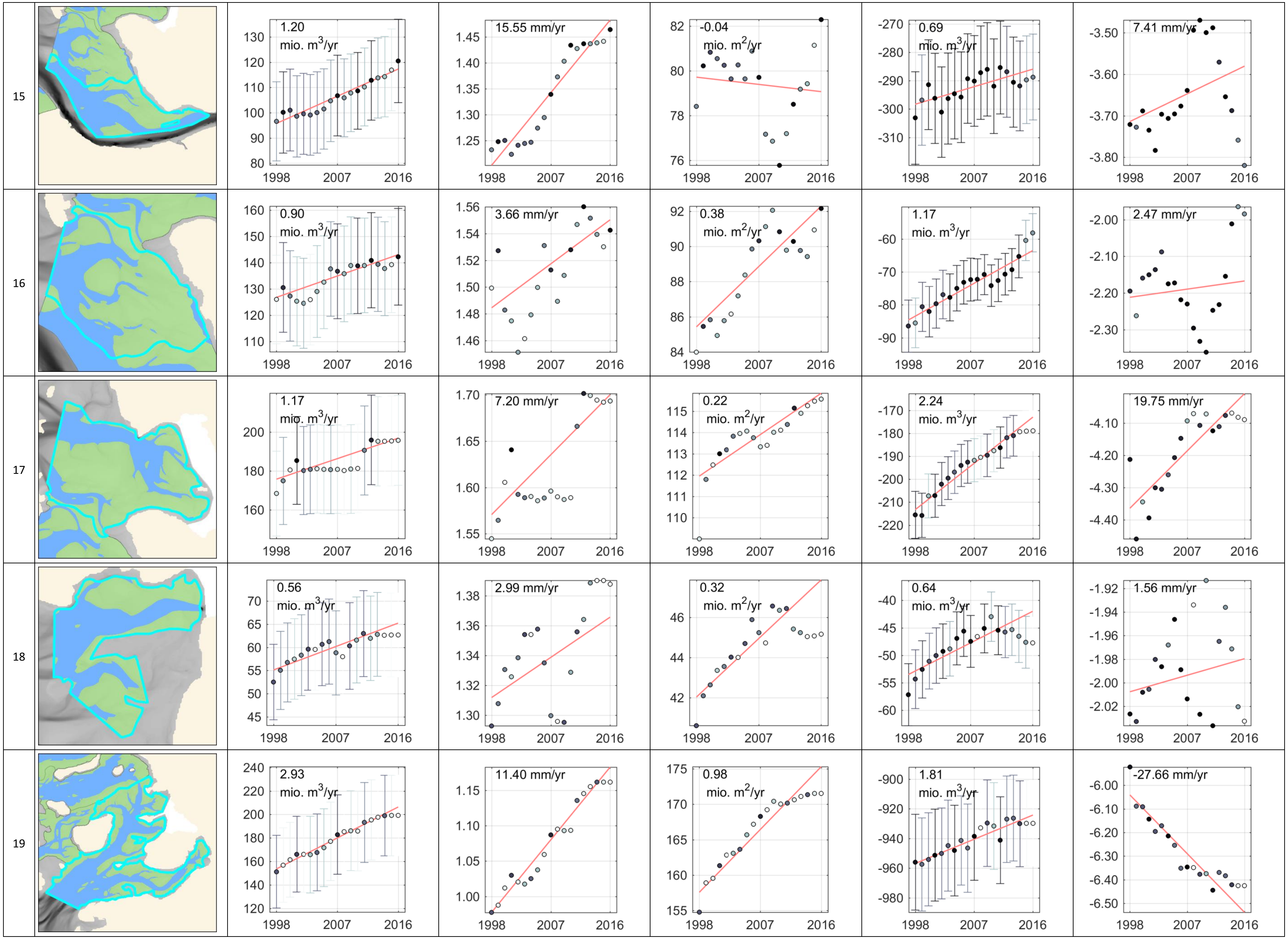
15
1.20 mio. m3/yr
15.55 mm/yr
-0.04 mio. m2/yr
0.69 mio. m3/yr
7.41 mm/yr
16
0.90 mio. m3/yr
3.66 mm/yr
0.38 mio. m2/yr
1.17 mio. m3/yr
2.47 mm/yr
17
1.17 mio. m3/yr
7.20 mm/yr
0.22 mio. m2/yr
2.24 mio. m3/yr
19.75 mm/yr
18
0.56 mio. m3/yr
2.99 mm/yr
0.32 mio. m2/yr
0.64 mio. m3/yr
1.56 mm/yr
19
2.93 mio. m3/yr
11.40 mm/yr
0.98 mio. m2/yr
1.81 mio. m3/yr
-27.66 mm/yr
1998
2007
2016

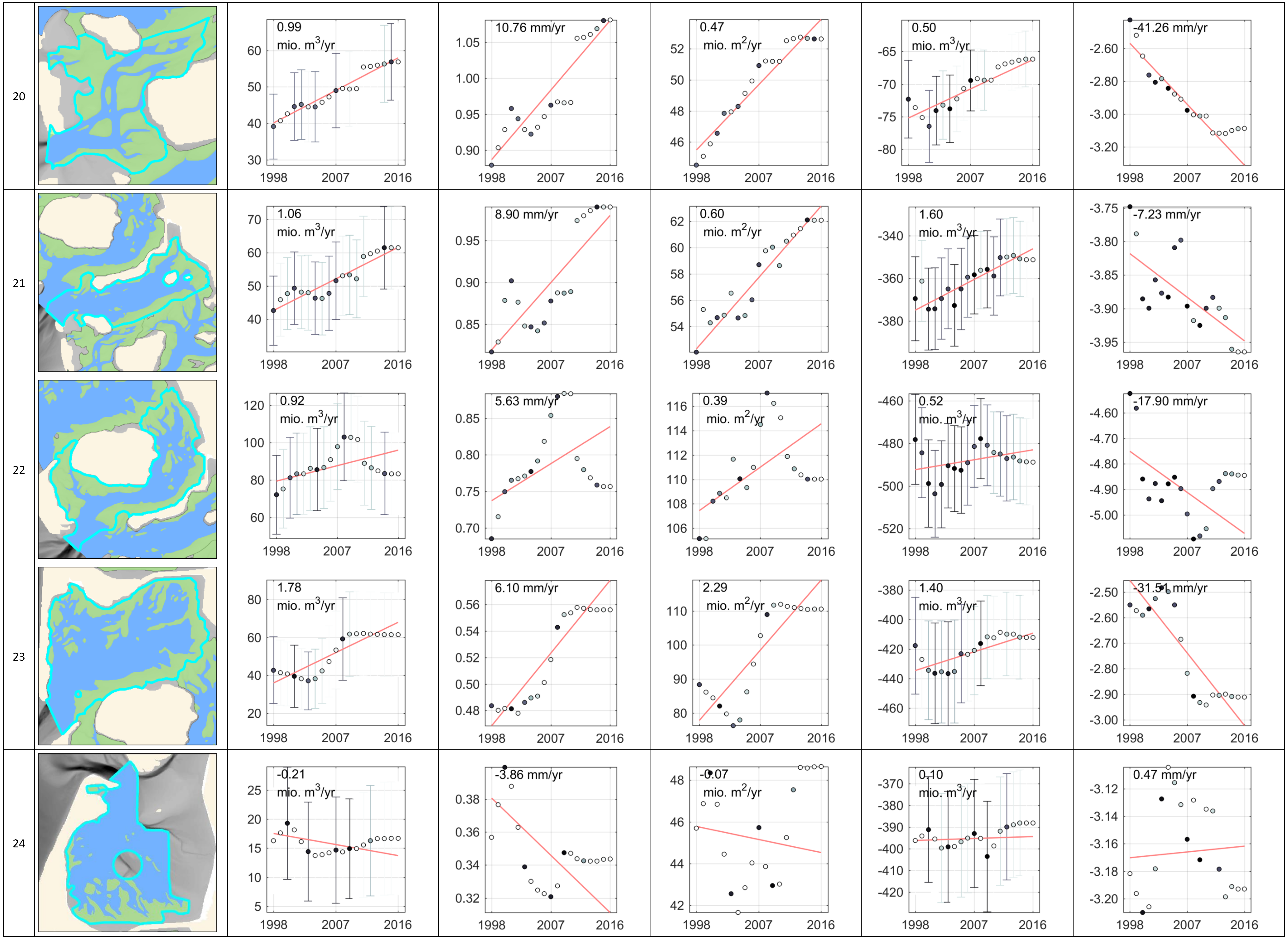
20
0.99
mio. m3/yr
10.76 mm/yr
0.47
mio. m2/yr
0.50
mio. m3/yr
-41.26 mm/yr
21
1.06
mio. m3/yr
8.90 mm/yr
0.60
mio. m2/yr
1.60
mio. m3/yr
-7.23 mm/yr
22
0.92
mio. m3/yr
5.63 mm/yr
0.39
mio. m2/yr
0.52
mio. m3/yr
-17.90 mm/yr
23
1.78
mio. m3/yr
6.10 mm/yr
2.29
mio. m2/yr
1.40
mio. m3/yr
-31.51 mm/yr
24
-0.21
mio. m3/yr
-3.86 mm/yr
-0.07
mio. m2/yr
0.10
mio. m3/yr
0.47 mm/yr
1998
2007
2016

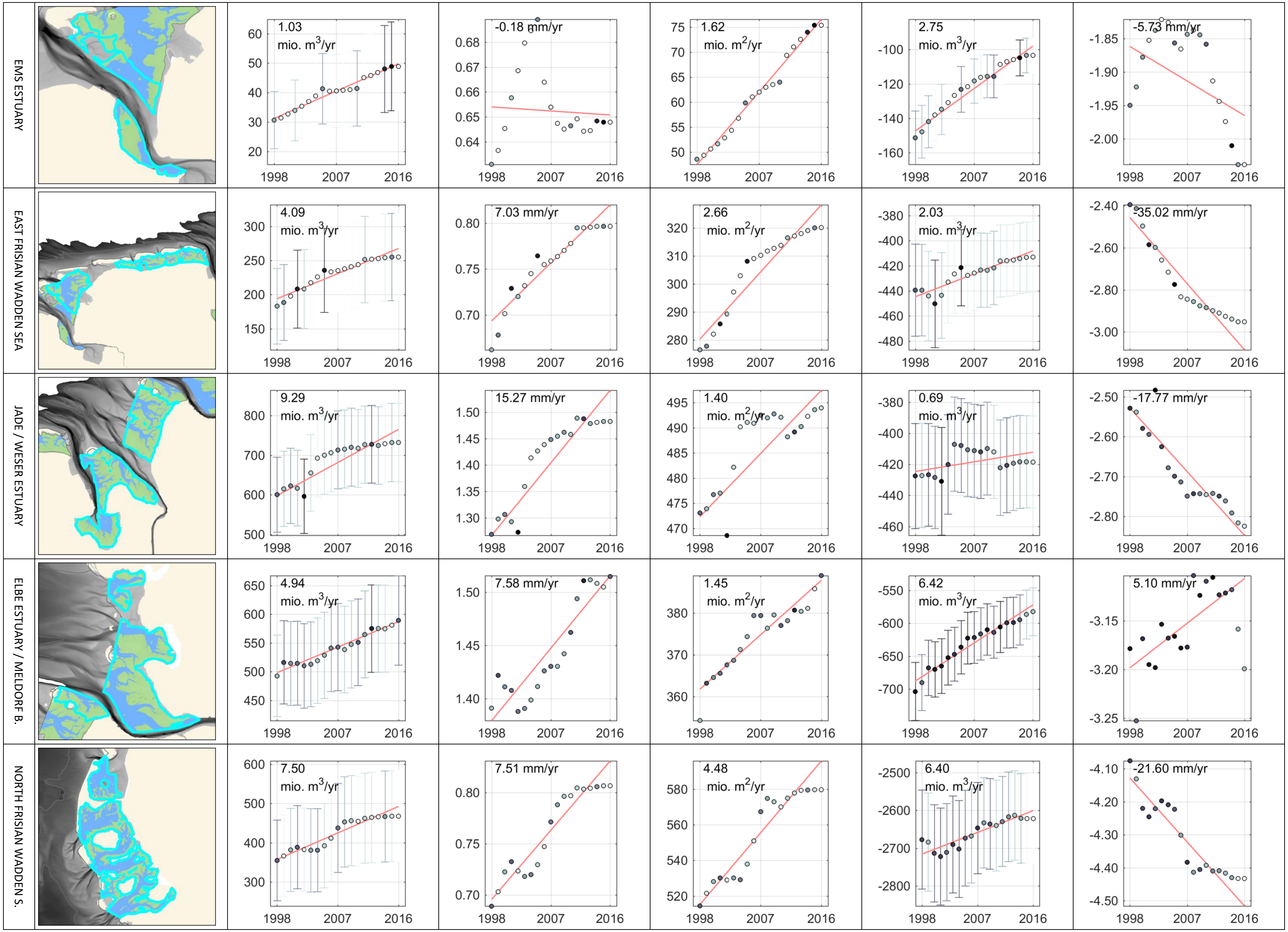

EMS ESTUARY
1.03 mio. m3/yr
-0.18 mm/yr
1.62 mio. m2/yr
2.75 mio. m3/yr
-5.73 mm/yr
EAST FRISIAN WADDEN SEA
4.09 mio. m3/yr
7.03 mm/yr
2.66 mio. m2/yr
2.03 mio. m3/yr
-35.02 mm/yr
JADE / WESER ESTUARY
9.29 mio. m3/yr
15.27 mm/yr
1.40 mio. m2/yr
0.69 mio. m3/yr
-17.77 mm/yr
ELBE ESTUARY / MELDORF B.
4.94 mio. m3/yr
7.58 mm/yr
1.45 mio. m2/yr
6.42 mio. m3/yr
5.10 mm/yr
NORTH FRISIAN WADDEN S.
7.50 mio. m3/yr
7.51 mm/yr
4.48 mio. m2/yr
6.40 mio. m3/yr
-21.60 mm/yr
1998
2007
2016
